# Hydro-ethanol extract of *Holarrhena floribunda* stem bark exhibits anti-anaphylactic and anti-oedematogenic effects in murine models of acute inflammation

**DOI:** 10.1186/s12906-022-03565-6

**Published:** 2022-03-19

**Authors:** Stephen Antwi, Daniel Oduro-Mensah, David Darko Obiri, Newman Osafo, Aaron Opoku Antwi, Helena Owusu Ansah, Augustine Ocloo, Laud K. N.-A. Okine

**Affiliations:** 1 Department of Pharmacology, Faculty of Pharmacy and Pharmaceutical Sciences, College of Health Sciences, Kwame Nkrumah University of Science and Technology, Kumasi, Ghana; 2 Pharmacology/Toxicology Department, Centre for Plant Medicine Research (CPMR), Mampong-Akuapem, Ghana; 3 Department of Biochemistry, Cell and Molecular Biology, School of Biological Sciences, College of Basic and Applied Sciences, University of Ghana, Legon, Ghana

**Keywords:** Anaphylaxis, COVID-19, Inflammation, Oedema, Prophylactic, Therapeutic

## Abstract

**Background:**

*Holarrhena floribunda* (G.Don) T.Durand & Schinz stem bark has anecdotal use in Ghanaian folk medicine for the management of inflammatory conditions. This study was conducted to investigate the in vivo anti-inflammatory activity of the bark extract using models of acute inflammation in male Sprague Dawley rats, C57BL/6 mice and ICR mice.

**Methods:**

A 70% hydro-ethanol extract of the stem bark (HFE) was evaluated at doses of 5–500 mg/kg bw. Local anaphylaxis was modelled by the pinnal cutaneous anaphylactic test. Systemic anaphylaxis or sepsis were modeled by compound 48/80 or lipopolysaccharide, respectively. Clonidine-induced catalepsy was used to investigate the effect on histamine signaling. Anti-oedematogenic effect was assessed by induction with carrageenan. Effects on mediators of biphasic acute inflammation were studied using histamine and serotonin (early phase) or prostaglandin E2 (late phase).

**Results:**

HFE demonstrated anti-inflammatory and/or anti-oedematogenic activity comparable to standard doses of aspirin and diclofenac (inhibitors of cyclooxygenases-1 and -2), chlorpheniramine (histamine H1-receptor antagonist), dexamethasone (glucocorticoid receptor agonist), granisetron (serotonin receptor antagonist) and sodium cromoglycate (inhibitor of mast cell degranulation). All observed HFE bioactivities increased with dose.

**Conclusions:**

The data provide evidence that the extract of *H. floribunda* stem bark has anti-anaphylactic and anti-oedematogenic effects; by interfering with signalling or metabolism of histamine, serotonin and prostaglandin E_2_ which mediate the progression of inflammation. The anti-inflammatory and antihistaminic activities of HFE may be relevant in the context of the management of COVID-19.

## Background

Inflammation serves as an immune response mechanism to rid the body of uncharacteristic stimuli due to invasion or injury. Several physiological pathways contribute to the immune inflammatory response. Dysfunction of the inflammatory response often leads to disability, organ malfunction, severe morbidity and even mortality if left untreated. Such dysregulation is what has been linked with fatality due to SARS-CoV-2 infection, resulting from a cytokine storm and subsequent systemic hyper inflammation [1–3]. Even before COVID-19, inflammation-associated chronic disease conditions had been listed as the leading threat to human health [4, 5]. Disease burden due to aberrant inflammation continues to increase, causing long-term morbidity and/or disability, and negatively affecting quality of life and economic well-being [6].

Anti-inflammatory agents used for treatment of inflammation-associated disease conditions include antihistamines, glucocorticoids, non-steroidal anti-inflammatory drugs, disease-modifying anti rheumatic drugs (DMARDs) and biologic drugs. These agents are used in different combinations for their distinct effects, a practice which highlights a significant challenge with management of inflammation-associated diseases — the fact that conventional anti-inflammatory agents typically have specific inflammation mediators as targets, leaving other uninhibited mediators to compensate. Furthermore, several of the agents have demonstrated significant toxicity in humans [7–11]. This makes them particularly unsuited for prophylactic or long-term use. There is the need to seek alternatives that have (1) potent activity, (2) non-toxicity to humans, particularly with prolonged use, and (3) ability to interact with multiple inflammatory response mediators.

Natural products, particularly from plant sources, continue to provide leads for development of medicinal compounds, including the majority of all medications in modern drug therapy [12–15]. *Holarrhena floribunda* is a shrub to medium-sized tree whose stem bark has popular anecdotal use in Ghanaian traditional settings for treatment of inflammation-related conditions. In other parts of West Africa, it is reported to be used medicinally as an anti-diabetic [16], antibacterial and antifungal [17] agent. This is the first in a series of three reports describing the anti-inflammatory bioactivity of the plant. Here, we describe the inhibitory effects of the hydro-ethanol extract of *H. floribunda* stem bark on acute inflammation. This study uses carrageenan/mediator-induced inflammation, and antigen-induced anaphylaxis, in murine models to highlight the broad-spectrum anti-inflammatory and antihistaminic activities of the plant part. Mediator-induced inflammation in rat and mouse models has predictive value for the assessment of anti-inflammatory agents that interact with inflammation mediators including histamine, bradykinin, serotonin, selected cytokines and tumor necrosis factor alpha [18–21]. Also, striking similarities in the serology of antigen-induced anaphylaxis between humans and laboratory rat and mouse models have been shown [22, 23].

## Methods

### Chemicals and Reagents

Compound 48/80, carrageenan, diclofenac, lipopolysaccharide (*Escherichia coli* O127:B8 LPS), aspirin and dexamethasone were purchased from Sigma-Aldrich (St Louis, USA). Clonidine was purchased from Boehringer Ingelheim Inc (Ridgefield, USA), chlorpheniramine was from DWD Pharmaceuticals Ltd (Mumbai, India) and haloperidol rom from Incas Pharmaceuticals Pt. Ltd (Ahmedabad, India). Bovine serum albumen (BSA) was obtained from PAA Laboratories (Marburg, Germany). Sodium cromoglycate was purchased from Ashford Lab Pt. Ltd, (Mumbai, India), granisetron hydrochloride from Roche (Basel, Switzerland) and phosphate buffered saline (PBS) from Gibco (Karlsruhe, Germany).

### Experimental animals

The protocols for this study, including all animal experiments, were approved by the Ethics Committee of Centre for Plant Medicine Research, Mampong-Akuapem (approval number CPMR/M.6-PT3/2018). The animals were handled in accordance with internationally accepted principles of laboratory animal use and care (EEC Directive 2010/63/EU). A total of 216 male Sprague Dawley rats (SDR, 200–220 g), 36 C57BL/6 mice and 36 ICR mice (25–30 g) were obtained from and maintained at the Animal Experimentation Unit of Centre for Plant Medicine Research (CPMR), Mampong-Akuapem, Ghana. The animals were kept under ambient laboratory conditions: temperature (28 ± 2) °C, relative humidity 60–70%, and a normal light dark cycle of 12 h. The animals were allowed access ad libitum to sterilized drinking water and powdered feed obtained from Ghana Agro Food Company (GAFCO), Tema. Animals were randomly assigned to groups labelled either as control (vehicle or positive) or extract treatment groups. Each group was housed in a metallic cage with dimensions 200 cm × 252 cm^2^ for rats and 125 cm × 65 cm^2^ for mice. Polyvinyl chloride plastic tubes and a plastic ball were provided in each cage as a source of environmental enrichment. All animals were acclimatized for seven days in the designated experimentation room before the start of experiments. The animals were trained to allow cooperation with restraint and other handling procedures. Animals in each single cage were considered as one experimental unit and received the same treatment. Drug/extract/vehicle was administered orally by gavage.

### Animal sampling and blinding

The rats and mice were randomly assigned to groups using the randomization function on Microsoft Excel 2010. These groups were also randomly assigned to treatments in each experimental set. Each experimental set consisted of a total of 30 animals placed in 5 groups of 6 animals. The sample size that was used was calculated using the statistical software G*power 3.1.9.2 with effect size of 0.85, α probability of type one error of 0.05, two tailed and a power of 80%. All animals were kept in the same controlled room at the same level on metallic shelves. On treatment days, animals were dosed in random order with drug/extract/vehicle. Investigators and technicians responsible for experimental procedures were blinded. One scientist prepared drugs/extracts and assigned alphabetical designations. Another scientist administered drugs/extract/vehicle randomly to groups while a third scientist was responsible for observations and/or assessment of indices of interest. Statistical analyses were performed by a scientist who was blinded to the data groupings.

### Humane endpoint and euthanasia

The behavior of animals used in the experiments was monitored hourly for 12 h after administration of drug/extract, and then subsequently observed at 12-h intervals. Body condition scoring [24] was used to monitor the health of animals. Modifications of general and social behaviour were used as proxy to indicate animals in pain and distress. It was considered that animals that were not well groomed, with awkward gait, slightly hunched and/or agitated when touched were in distress. Animals were placed in social groups and trained before all experimental procedures. In all experiments where anaphylactic reactions and/or oedema were expected, and also where death of animals was necessary, humane endpoints were adopted to alleviate pain and distress, as well as euthanasia where necessary. The humane endpoints considered body temperature below 34 °C, labored respiration evidenced by excessive abdominal involvement, reduced exploration, reduced grooming, inability to access food and water, and lack of response to manipulation [25, 26]. Euthanasia methods used were 800 mg/kg *i.p.* administration of pentobarbital sodium [27] in the case of rats and cervical dislocation in mice.

### Preparation of Hydro-ethanol Extract of *H. floribunda*

*H. floribunda* stem bark was obtained from the wild in Kwahu-Asakraka (6º38’02.6”N;0º41’37.5”W), Ghana. The plant part was collected, identified and authenticated by Mr. Herone Blagogee (Research Officer/Botanist) of the Plant Development Department of CPMR. The Plant Development Department is licenced by the Forest Services Division of Forestry Commission of Ghana to source for plant material from the arboretum of CPMR and the wild. A specimen with voucher number 05/13 has been kept at the herbarium of CPMR. The material was washed, chopped into pieces, air-dried, and milled into a coarse powder. For extraction, 1 kg of powdered stem bark was macerated in 5 L ethanol (70% v/v) with periodic stirring, decanted after 72 h and filtered. Ethanol was removed by rotary evaporation (EYELA, Shanghai, China) and the aqueous concentrated extract was lyophilised to obtain powder with a yield of 7.33% w/w. This was subsequently referred to as hydro-ethanol extract of *H. floribunda* (HFE). The lyophilate was reconstituted in normal saline (0.9% w/v NaCl) for use in subsequent assays.

### Determination of Median Lethal Dose, LD_50_

A single dose *p.o.* of 5000 mg/kg HFE was administered to SDRs (*n* = 6) and ICR mice (*n* = 6). The animals were observed over a 48-h period for general behaviour and euthanized at humane endpoints to prevent pain and distress. Animals surviving beyond 48 h were observed further over 12 days for signs of toxicity: piloerection, lachrymation, and difficulty with movement or breathing.

Subsequently, five murine models of acute inflammation were used to investigate the in vivo anti-inflammatory activity of HFE as described below.

### Anti-anaphylactic activity

#### Cutaneous anaphylaxis

The pinnal inflammation model [28] was adopted. Six groups (*n* = 6) of ICR mice were injected *s.c* with 100 µl of 0.05 mg/ml BSA at start of the experiment, and again after 14 days with 100 µl of 0.02 mg/ml BSA. On day 21, test mice received *p.o.* 0.3 mg/kg dexamethasone, 100 mg/kg aspirin or HFE (50, 200 or 500 mg/kg), respectively, while control mice received 0.1 ml normal saline. One hour after drug/extract administration, each mouse was put under isoflurane-induced anaesthesia (4%, drop jar method), 200 µl Evans Blue dye (1% w/v) was injected into the tail vein and both pinnae were immediately injected with 0.1 mg/ml BSA. Mice were euthanized 30 min later and their ears were cut off. Area of reaction was measured by circumscribing the area of extravasation of Evans blue dye and matching with the best fit of standard circles. Percentage inhibition of the inflammatory reaction was expressed as:

1 $$\% \,inhibition\,of\,reaction = 100\left( {\frac{{A_{t} - A_{o} }}{{A_{o} }}} \right)$$

where *A*_*o*_ and *A*_*t*_ are the area of extravasation of dye in the pinnae of saline (vehicle) control or drug/extract-treated mice respectively.

#### Compound 48/80-induced anaphylactic shock

Anaphylactic shock was induced by compound 48/80 [29]. C57BL/6 mice in 5 groups (*n* = 6) received *p.o.* 10 ml/kg saline (vehicle), 50 mg/kg sodium cromoglycate or HFE (50, 200 or 500 mg/kg) 1 h before administration of compound 48/80 (8 mg/kg, *i.p.*). Survival rate was monitored for 1 h post administration. Mice were euthanized at humane endpoints to prevent pain and distress and the time of euthanasia was used as the end point of the experiment.

#### Septic shock model

Septic shock was induced by LPS administration [30]. Groups of SDRs (*n* = 6) were challenged *i.p*. with 5 mg/kg LPS in saline at 10 ml/kg bw. In the prophylactic model, treatment was administered twice; 24 h and 1 h before LPS challenge. In the therapeutic model, treatment was only administered 1 h after LPS challenge. Treatment agents were administered *i.p.*: 10 ml/kg saline, 0.3 mg/kg dexamethasone or HFE (50, 200 or 500 mg/kg). Survival rate was monitored for 48 h after LPS challenge. Animals that showed no distress after 48 h were monitored for up to 168 h. Rats were euthanized at humane endpoints to prevent pain and distress and the time of euthanasia was used as the end point of the experiment.

#### Membrane stabilisation assay

Whole blood was collected from rats under 4% isoflurane anaesthesia into heparinised vacutainer tubes (Thomas Scientific, Swedesboro, NJ). The blood was washed (3000 rpm for 10 min) three times with 0.9% saline and reconstituted as a 40% v/v suspension with isotonic buffer (10 mM sodium phosphate buffer with 0.9% NaCl) at pH 7.4. Protection of red blood cells from heat-induced hemolysis was assessed as described by [31, 32]. Absorbance measurements were taken at 540 nm using Shimadzu UV-160A spectrophotometer. Acetyl salicylic acid (ASA) 200 µg/ml was used as a reference standard. Percent inhibition of hemolysis was calculated as:

2 $$\% \,inhibition\,of\,hemolysis = 100 \times \left( {1 - \frac{{OD_{2} - OD_{1} }}{{OD3 - OD_{1} }}} \right)$$

where OD_1_, OD_2_ and OD_3_ are absorbance readings for unheated test sample, heated test sample and heated control sample, respectively.

#### Clonidine-induced catalepsy

Indirect antihistaminic activity was investigated [33]. Groups of ICR mice (*n* = 6) were treated *p.o.* with 10 ml/kg saline, 4 mg/kg chlorpheniramine or HFE (50, 200 or 500 mg/kg). In the prophylactic protocol, control drug or extract was administered 1 h prior to catalepsy induction while in the therapeutic protocol, drug or extract was administered 1 h post induction. Animals received 5 mg/kg clonidine *s.c*. and were made to grip a horizontal bar (1 cm diameter, 3 cm high) with their fore paws. The time taken to let go was recorded as duration of catalepsy, measured at 30 min interval for 3 h after catalepsy induction. Maximal cataleptic effect was estimated by change in catalepsy calculated as: Treated mice were euthanized at humane endpoints to prevent pain and distress

3 $$\% \;Change\;in\,catalepsy = 100 \times \left( {\frac{{Catalepsy\left( {T_{t} } \right) - Catalepsy\left( {T_{0} } \right)}}{{Catalepsy\left( {T_{0} } \right)}}} \right)$$

where *T*_*t*_ and *T*_*0*_ are catalepsy at a time point of measurement and catalepsy at baseline, respectively.

Total catalepsy induced was measured as area under the time course curve (AUC).

### Anti-inflammatory Activity: Paw Oedema in Mice

Paw oedema was induced in groups (*n* = 6) of SDRs by sub-plantar injection of 100 µl of a phlogistic agent (1% w/v) in the right hind paw [34, 35]. Phlogistic agents used were carrageenan, histamine, serotonin or prostaglandin E_2_, all freshly prepared in normal saline. In the prophylactic protocol, 10 ml/kg saline, 4 mg/kg chlorpheniramine, 100 µg/kg granisetron, 100 mg/kg diclofenac or HFE (50, 200 or 500 mg/kg) were administered *p.o*. before injection of the phlogistic agent. In the therapeutic protocol, control drug or extract was given 1 h after injection of carrageenan. Paw volumes were measured by a plethysmometer at 1 h intervals for 4 h. Raw measures for paw volume were normalised as percentage change from the baseline value. Maximal oedema response was calculated as:

4 $$\% \;Increase\;in\;paw volume = 100 \times \left( {\frac{{pv_{t} - pv_{o} }}{{pv_{o} }}} \right)$$

where pv(t) and pv(0) are paw volume at a point of measurement and at baseline, respectively.

Total oedema induced over the 4 h period was measured as area under the time course curves (AUC).

### Statistical analyses

Data were analysed by one-way analysis of variance (ANOVA), followed by Dunnett’s multiple comparison post hoc test on GraphPad Prism for Windows Version 5.00 (GraphPad, San Diego, CA). Data are expressed as mean ± standard error of mean. Survival curves were analysed by Log-rank (Mantel Cox) test. All data from experimental units were included in data analysis. Each analysis reported had 6 values of specific outcome parameters obtained from 6 animals in an experimental unit.

## Results

### LD_50_ of HFE

No mortality was recorded within 48 h after administration of a single oral dose of HFE (5000 mg/kg bw). There were no physical signs of toxicity, as evidenced by normal locomotory and respiratory activity. No lachrymatory effect, bulging of eyes or piloerection was observed.

### Anti-anaphylactic Activity of HFE

#### Cutaneous anaphylaxis

**Fig. 1:**
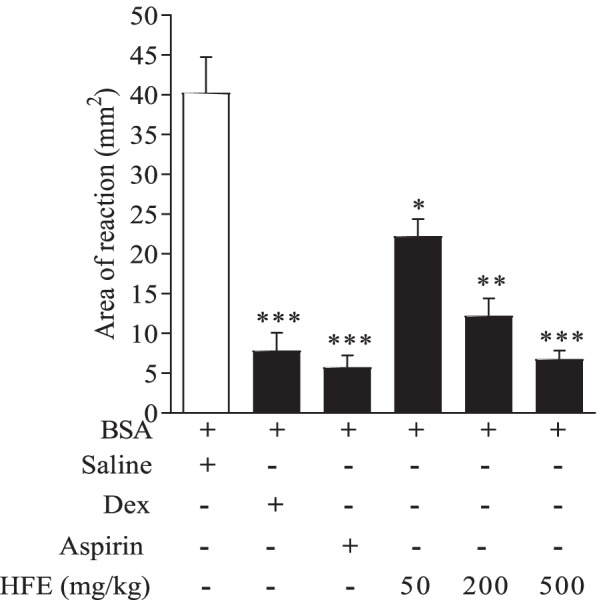
Inhibition of pinnal cutaneous anaphylaxis by *Holarrhena floribunda* stem bark extract in ICR mice. Sensitized mice received normal saline 10 ml/kg, aspirin 100 mg/kg, dexamethasone 0.3 mg/kg or HFE 50–500 mg/kg, *p.o*. Data were analysed by one-way ANOVA followed Dunnet’s post hoc test, and are presented as mean ± S.E.M of *n* = 5. Significance is relative to saline-treated control. ^*^P < 0.01, ^**^P < 0.001, ^***^P < 0.0001

Pinnal challenge with BSA in previously sensitized mice induced local inflammation marked by extravasation of Evans blue dye. Mean reaction area for the saline-treated control was 40.25 ± 4.52 mm^2^ (Fig. 1). HFE reduced area of extravasation to 6.77–22.26 mm^2^ (*P* ≤ 0.013). The reduction by 500 mg/kg HFE was comparable (*P* ≥ 0.809) to the values for dexamethasone (7.85 mm^2^) and aspirin (5.75 mm^2^).

#### Compound 48/80-induced shock

**Fig. 2:**
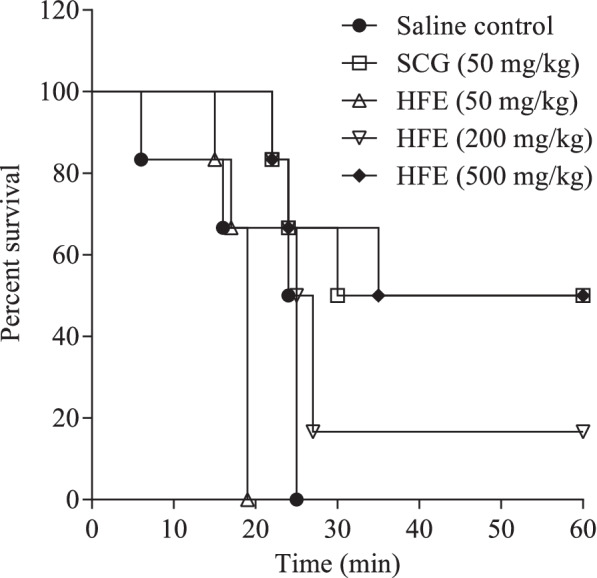
Effect of *Holarrhena floribunda* stem bark extract on compound 48/80-induced systemic anaphylaxis. C57BL/6 mice received normal saline 10 ml/kg, sodium cromoglycate (SCG) 50 mg/kg or HFE 50–500 mg/kg *p.o.* 1 h prior to challenge. Data (*n* = 6) were analysed by Log-rank (Mantel Cox) test. Differences between the control plot and survival plots for treatment groups were significant (*P* ≤ 0.001) between 20–60 min

Compound 48/80 caused 100% mortality within 25 min of administration in all saline-treated control mice (Fig. 2). Survival proportion for sodium cromoglycate was 50% over 60 min. HFE protected mice, evidenced by the survival proportions increasing from 0 (50 mg/kg) to 16 or 50% for the 200 or 500 mg/kg groups, respectively.

#### Lipopolysaccharide-induced septic shock

**Fig. 3:**
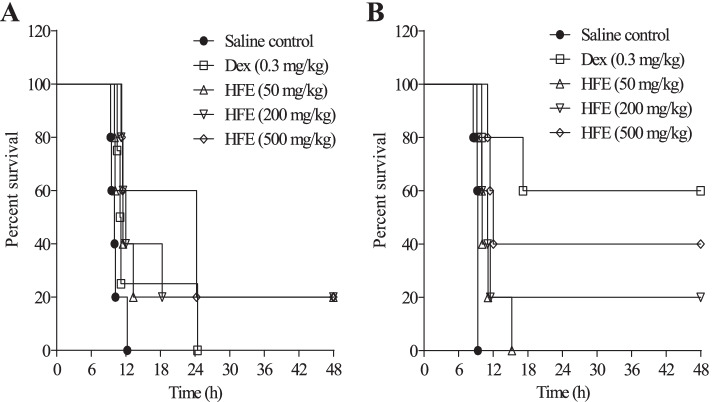
Protection by *Holarrhena floribunda* stem bark extract from LPS-induced anaphylaxis. Sprague–Dawley rats received saline 10 ml/kg, dexamethasone 0.3 mg/kg or HFE 50–500 mg/kg, either prophylactic (**A**) or therapeutic (**B**). Data (*n* = 6) were analysed using Log-rank (Mantel Cox) test. Differences in survival curves between the control and HFE treatment groups were significant for prophylactic HFE administration at 500 mg/kg (*P* ≤ 0.0198) and all therapeutic HFE doses (*P* ≤ 0.0412–0.0034)

In septic shock modelled by LPS-induced anaphylaxis, 100% mortality was observed among saline-treated control mice 10–12 h after LPS challenge. In the prophylactic model, drug/extract-treated rats survived beyond 12 h (Fig. 3A). Dexamethasone failed to protect beyond 24 h whereas survival proportion for all HFE-treated groups was 20%. Therapeutic HFE administration protected rats, with survival proportions of 0, 20 or 40% for the 50, 200 or 500 mg/kg groups, respectively (Fig. 3B). Survival for therapeutic dexamethasone was 60%.

### Membrane stabilisation assay

**Fig. 4:**
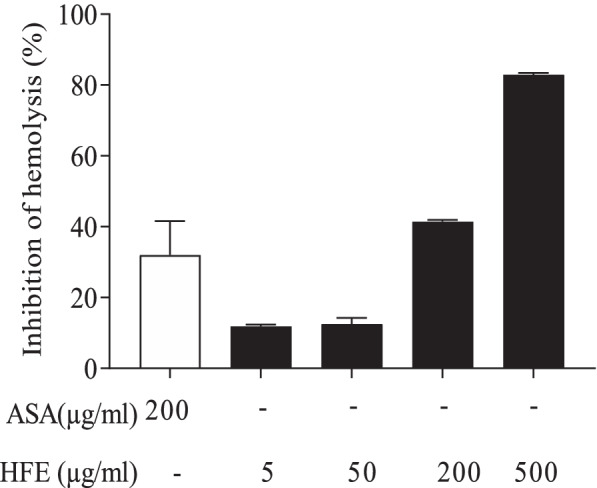
*Holarrhena floribunda* stem bark extract protects rat erythrocytes from heat-induced haemolysis. Whole blood was collected from rats under anaesthesia, washed and reconstituted as a 40% v/v red blood cell suspension. Red blood cell suspensions were incubated with HFE (5, 50, 200 or 500 µg/ml). Acetyl salicylic acid (ASA) 200 µg/ml was used as reference. Absorbance measurements were taken at 540 nm. Data for percent inhibition of haemolysis are presented as mean ± SEM of *n* = 4

HFE protected rat erythrocyte membrane from heat-induced lysis (Fig. 4). HFE at 200 μg/ml had activity comparable to 200 µg/ml acetylsalicylic acid (reference drug), whereas 500 μg/ml HFE had approximately twice the activity of the reference.

### Clonidine- and haloperidol-induced catalepsy

**Fig. 5:**
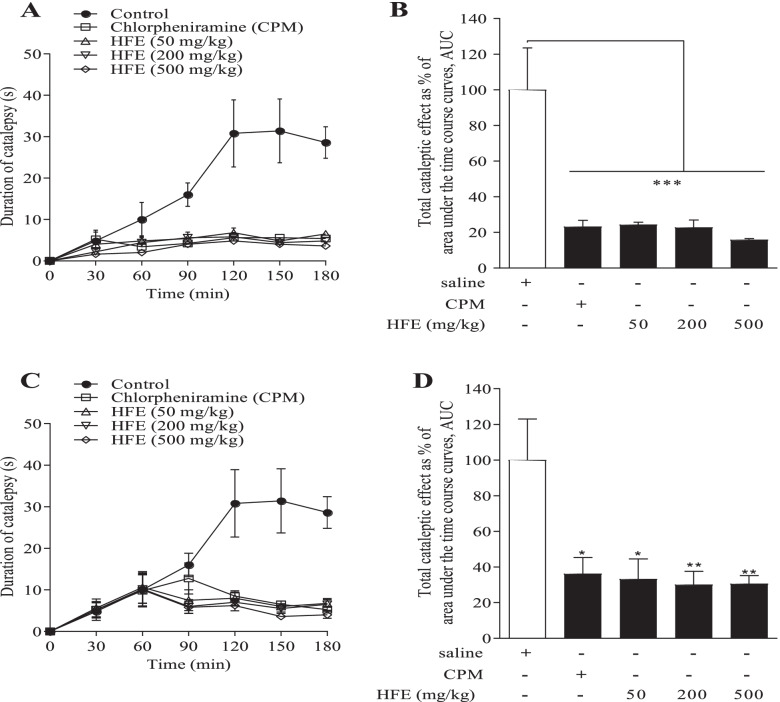
*Holarrhena floribunda* stem bark extract protects from clonidine-induced catalepsy. ICR mice received saline 10 ml/kg, chlorpheniramine (CPM) 4 mg/kg or HFE 50–500 mg/kg prophylactic (**A**, **B**) or therapeutic (**C**, **D**). Total catalepsy was calculated as area under the time course curves (right panel). Data were analysed by one-way ANOVA followed Dunnet’s post hoc test, and are presented as mean ± SEM of *n* = 6. Indications of significance are relative to the saline-treated control. *P < 0.05, **P < 0.01, ***P < 0.001, ns is not significant

Relative to the saline-treated control (31.4 ± 8.1 s), prophylactic HFE reduced maximum duration of clonidine-induced catalepsy to 4.8–6.8 s, *P* ≤ 0.001 (Fig. 5A). Total catalepsy was also reduced to 15.8–24.4% (*P* ≤ 0.002) of the control value (Fig. 5B). Therapeutic administration of HFE reduced maximum duration of clonidine-induced catalepsy from the control value of 19.0 ± 4.5 s to 6.2–8.5 s, *P* ≤ 0.047 (Fig. 5C). Again, total catalepsy was reduced to 29.8–38.5% (*P* ≤ 0.044) of the control value (Fig. 5D).

### HFE anti-oedematogenic activity

#### Carrageenan-induced paw oedema

Paw oedema peaked between 2–3 h in control rats (Figs. 6.1 A and 6.1 C). In the prophylactic model, percentage mean maximal oedema for the saline-treated control group was 67.7 ± 9.1% of baseline value (Fig. 6.1 A). HFE caused reductions to 33.4–46.6%, which were significant (*P* ≤ 0.0015) for the 200 and 500 mg/kg doses. Maximal oedema for diclofenac was 6.29% of baseline value, and total paw oedema over the 4 h period was reduced to 10.9% of the control value (Fig. 6.1 B). Reduction of oedema by HFE was significantly different (*P* ≤ 0.02) from the control only for the 200 (39.0%) and 500 mg/kg (34.6%) doses.

On therapeutic administration, percentage mean maximal oedema for the inflamed control group was 59.2 ± 7.6% of baseline (Fig. 6.1 C). HFE reduced the mean maximal oedema to 31.2–42.6% (*P* ≤ 0.04). Total paw oedema over the 4 h period was reduced to 43.4–66.6% of the control value (Fig. 6.1 D). Reduction by 200 and 500 mg/kg HFE were different (*P* ≤ 0.004) from the inflamed control value and were comparable (*P* ≥ 0.99) to reduction by diclofenac.

#### Mediator-induced oedema

Percentage mean maximal histamine-induced oedema for the saline-treated control was 70.9 ± 4.5% of baseline (Fig. 7A). HFE caused a reduction to 46.9–54.5%. Total paw oedema was reduced to 65.1–77.9% of the inflamed control (Fig. 7B), significant only for 200 and 500 mg/kg HFE (*P* ≤ 0.022). Inhibition of total oedema by chlorpheniramine (64.9%) was not significantly different (*P* ≥ 0.54) from inhibition by HFE.

Maximal oedema due to serotonin was 59.2 ± 8.08% for the control group. HFE reduced the percentage mean maximal oedema to 15.8–28.2% (*P* ≤ 0.003) of baseline value (Fig. 7C). Total paw oedema was also reduced to 19.2–48.9% (*P* ≤ 0.003) of the control value (Fig. 7D). Inhibition of oedema by HFE was not different (*P* ≥ 0.38) from inhibition by granisetron (27.3%).

Prostaglandin E_2_ produced mean maximal oedema 75.4 ± 1.5% of the baseline value, which was reduced by HFE to 16.5–32.8%, *P* ≤ 0.001 (Fig. 7E). Total paw oedema also reduced to 21.6–45.6% (*P* ≤ 0.001) of the control value (Fig. 7F). Inhibition by diclofenac (10.1%) was not different (*P* ≥ 0.15) from inhibition by 200 and 500 mg/kg HFE.

## Discussion

This report highlights the broad-spectrum anti-inflammatory activity of the hydroethanolic extract of *H. floribunda* stem bark (HFE). Different rodent models were used to represent anaphylaxis and oedema due to distinct mechanisms of immune inflammatory response. In the cutaneous anaphylaxis design used in this study, the local allergic response to BSA is thought to be a type I hypersensitivity response induced mainly by vasoactive and pro-inflammatory mediators, primarily histamine released from mast cells and basophils [36–39]. Like localized anaphylaxis, systemic anaphylaxis due to LPS and compound 48/80 are also mediated through degranulation of mast cells [40–43], followed by perturbation of the membrane of other immune cells which allows inflammation mediators to leak out into circulation [44]. HFE reduced cutaneous anaphylaxis (Fig. 1), and protected mice from mortality due to systemic anaphylaxis or septic shock from compound 48/80 (Fig. 2) or LPS (Fig. 3), respectively. Upon challenge with compound 48/80 which is potent at causing mast cell degranulation [40, 44, 45], HFE at 500 mg/kg offered better protection than sodium cromoglycate (50 mg/kg) in terms of survival duration (Fig. 2) and was observed to delay onset of symptoms of shock and anaphylaxis. Together, these suggest that HFE may either be acting as (1) a mast cell membrane stabilizer, similar to sodium cromoglycate, to inhibit release of inflammation mediators and/or (2) an antagonist of histamine and/or other inflammation mediators released after mast cell degranulation.

LPS-induced anaphylaxis is mediated in its early stages by cytokines, including IL-1α, IL-6 and IL-10, all of which have short half-lives [46–48]. This is followed by stimulation of mast cell degranulation after approximately 6 h of challenge [49]. Data from this anaphylaxis model (Fig. 3) gave indication of the ability of HFE to interact with the indicated cytokines and added to the evidence of HFE interaction with mediators released from mast cells. Mediators released from mast cells are critical to anaphylactic shock, possibly explaining why mortality was not recorded in this experiment until after the 6th hour of LPS challenge (Fig. 3a & b). Whereas therapeutic administration of dexamethasone offered the highest level of protection up to 48 h, relative to the HFE doses, prophylactic administration (Fig. 3a) failed to protect beyond 24 h. Prophylactic administration of HFE afforded better protection than dexamethasone in terms of duration of survival throughout the 48-h observation period, as well as overall survival rate. When administered 1 h prior to challenge in the prophylactic model, blood dexamethasone levels may have peaked and began to drop even before LPS challenge, leaving less than the concentrations required for protective inhibition of expression/release of inflammation mediators [50, 51]. In the therapeutic model (Fig. 3b), blood dexamethasone levels would have peaked approximately 90 min after challenge. Therefore, cytokine involvement may already have occurred by the time the treatment agent was introduced, and the inflammatory response would have started shifting toward mediator synthesis and degranulation. Dexamethasone is known to strongly inhibit histamine synthesis [52, 53]. This may explain the high rate of protection seen with dexamethasone in the therapeutic model but not the prophylactic. The low levels of dexamethasone available after challenge in the prophylactic model may not have interfered enough with the early-stage cytokine activity or inhibited histamine synthesis enough to protect significantly from events post challenge. Unlike dexamethasone, any clearance of drug/extract that may have occurred did not significantly affect the protective effect of HFE in prophylactic administration since survival rate was similar for all HFE prophylactic doses. Protection due to therapeutic administration of HFE (200 and 500 mg/kg) was not significantly different (*p* = 0.5356 and 0.1740, respectively) from that of dexamethasone. It has been demonstrated in a rat model that the pharmacodynamics of dexamethasone is not different between arthritic and healthy rats [54]. Therefore, the dynamics of protection offered by dexamethasone in this study is unlikely to be attributable to adjusted physiology in the arthritic state.

Anti-inflammatory agents may act to inhibit release of mediators, including histamine, from proinflammatory cells. This is achieved by causing increase in the surface area volume ratio of cells through expansion of the membrane, shrinkage of the cell, interacting with membrane proteins or altering influx of calcium into the cells [40, 55, 56]. In the membrane protection/stabilization assay, HFE demonstrated activity comparable to an equal concentration of acetyl salicylic acid (Fig. 4). This suggests that HFE has potent membrane-stabilizing activity and may be capable of interfering with mast cell degranulation.

Ability of HFE to affect histamine metabolism is again indicated by the reduction in catalepsy observed upon HFE administration in clonidine-induced catalepsy (Fig. 5a-d). Unlike other catalepsy-inducing agents such as haloperidol [57], clonidine stimulates histamine release from mast cells, in a manner similar to compound 48/80 [58, 59]. The various stages of clonidine-induced catalepsy have been established to parallel brain levels of histamine [60], and the catalepsy may be inhibited by histamine receptor 1 (H1R) but not H2R antagonists [59, 61]. Again, mediator-induced edema due to histamine was inhibited by HFE (Fig. 7a), further demonstrating the interaction between HFE and histamine metabolism. It was expected that HFE effect on histamine-induced oedema would be stronger than was observed in this study. The moderate inhibition of oedema, however, supports the thinking that HFE effect on histamine metabolism may not only be post-degranulation but may involve suppression of production of the mediator.

Taken together, the strong HFE inhibitory activity on LPS-induced septic shock, the strong membrane-stabilizing activity and data from the histamine-induced oedema experiment suggest that (1) HFE may interact with mediators of early stage LPS-induced anaphylaxis, such as IL-1β, tumor necrosis factor alpha (TNF-α) and IL-6; (2) even if HFE does not inhibit histamine synthesis, it is be capable of inhibiting mast cell degranulation by membrane-stabilizing activity; and (3) HFE may be interfering with histamine signaling pre and post degranulation, and interacting with histamine receptor H1R, as well as H4R which has been shown to be a mediator in LPS-induced inflammation [62]. In addition, we note that before the 24 h time point, even the lowest prophylactic HFE dose offered better protection from LPS than dexamethasone, indicating better suitability for prophylactic use. These properties make HFE a potential candidate for use in management of COVID-19-associated inflammation.

Among other cytokines and inflammatory mediators, IL-1, -6, TNF-α and histamine have been strongly linked to COVID-19 severity and associated mortality [63–66]. Histamine plays a central role in acute inflammation and anaphylaxis [67, 68]. Relevant to COVID-19, histamine release from mast cells is known to mediate bronchoconstriction [69–72], and has been implicated in the progression of oedema observed in COVID-19 pulmonary disease [63]. Further supporting evidence of the primary role of histamine in COVID-19 progression include early symptoms such as anosmia, ageusia, skin rashes, neuropsychiatric symptoms, silent hypoxia and cachexia, all of which are consistent with dysfunctional histamine signaling [73–75]. In Ghana, the most common symptoms include cough, headache, sore throat, myalgia and anorexia (E. Oduro-Mensah, personal communication, August 14, 2020), also linked to elevated histamine levels. Interfering with histamine signaling after virus infection may provide a means to inhibit progression of disease and modulate the immune response to prevent mortality or severe morbidity from hyperinflammation [76]. In addition, the use of antihistamines can help reduce SARS-CoV-2 transmission through droplets since antihistamines may suppress symptoms such as sneezing and coughing [77]. With its demonstrated antihistamine activity, prophylactic or therapeutic HFE administration may contribute to inhibition of disease progression after SARS-CoV-2 infection and reduction of spread. This would be particularly useful in a low-income country like Ghana where most people already depend on plant-based remedies for management, treatment and/or prevention of disease, including COVID-19.

**Fig. 6:**
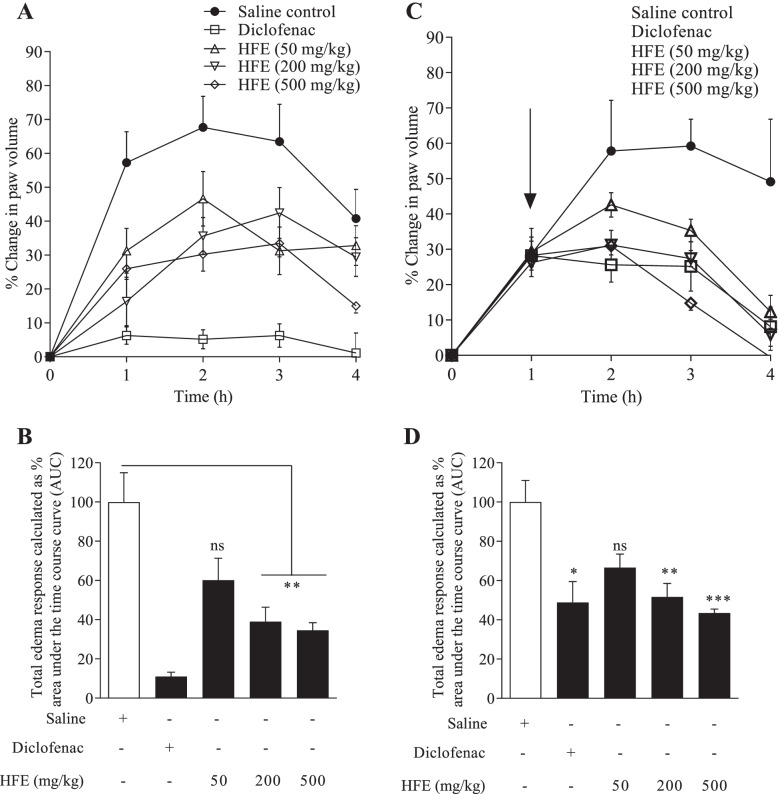
*Holarrhena floribunda* stem bark extract reduces carrageenan-induced paw oedema in mice. ICR mice received saline 10 ml/kg, diclofenac 4 mg/kg or HFE 50–500 mg/kg prophylactic (left panel) or therapeutic (right panel). Total oedema was calculated as area under the time course curves (B and D). Data are presented as mean ± S.E.M for *n* = 6. Arrow indicates point of extract administration in the therapeutic protocol. Indications of significance are relative to the saline-treated control. *P < 0.05, **P < 0.01, ***P < 0.001

**Fig. 7:**
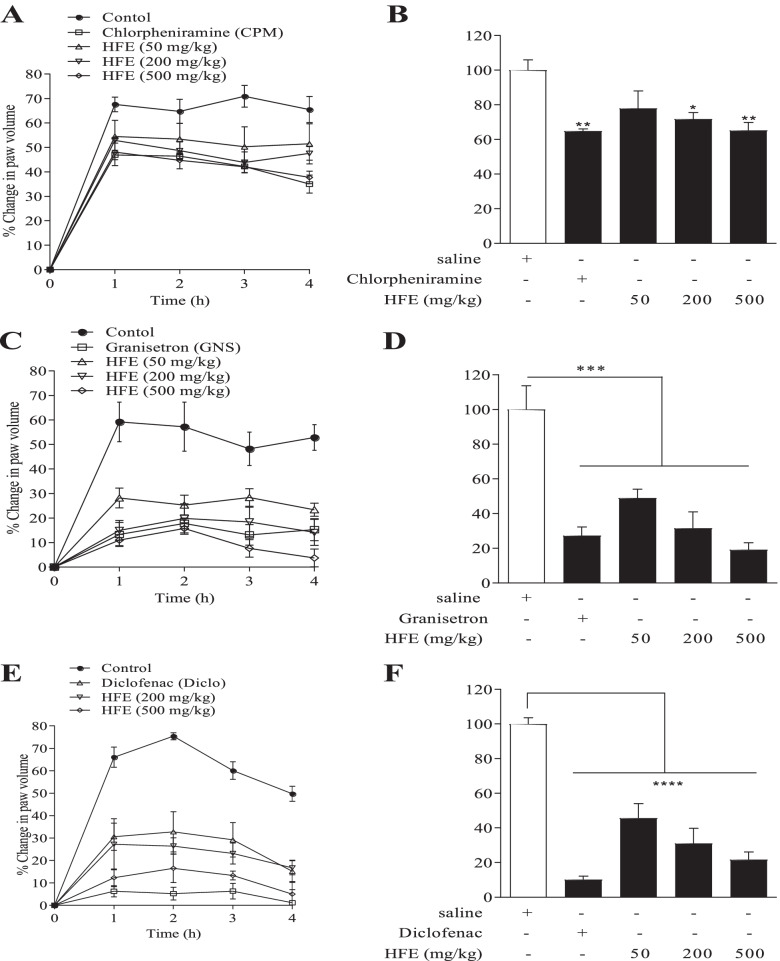
Effect of *Holarrhena floribunda* stem bark extract on mediator-induced paw oedema in mice. Mice received normal saline 1 ml/kg, chlorpheniramine 10 mg/kg, granisetron 100 µg/kg, diclofenac 0.93 mg kg or HFE 50–500 mg kg *p.o*. Total oedema was calculated as area under the time course curves (right panels). Data are presented as mean ± S.E.M of *n* = 6. Indications of significance are relative to the saline-treated control. *P < 0.05, **P < 0.01, ***P < 0.001, ****P < 0.0001

Progression of oedema after administration of carrageenan is a biphasic event. In this study, however, only the initial acute phase [78] whose mediators include histamine, serotonin, kinins, leukotrienes, cytokines and inducible cyclooxygenase (COX)-2 [20, 21, 79–82] was monitored. Diclofenac which was used as the reference agent is considered a broad-spectrum NSAID with multiple modes of action [83], and is known to be a strong inhibitor of COX-1 and -2 among others [84, 85]. The anti-inflammatory effect of HFE is evident from the oedema suppression in both the prophylactic and therapeutic models. Diclofenac administration as a prophylactic agent inhibited oedema by approximately 90% whereas HFE inhibited by up to 65% (Fig. 6a). In the therapeutic model, maximum oedema inhibition by HFE was similar to inhibition by diclofenac (48%) (Fig. 6b). Also, HFE strongly inhibited edema due to serotonin (Fig. 7b) and prostaglandin E2 (Fig. 7c), demonstrating its ability to interact with multiple inflammation mediators released from immune cells.

In this study, HFE has demonstrated both prophylactic and therapeutic anti-inflammatory effects. Particularly in relation to carrageenan-induced inflammation, this is significant due to a number of previous paradoxical observations indicating that prophylactic inflammation inhibitory effect of an agent does not necessarily imply therapeutic effect and vice versa [86, 87]. This study was limited to the use of different rodent models to represent anaphylaxis and oedema due to distinct mechanisms of immune inflammatory response in humans. Though the models provide useful information with regards to the effects of HFE on acute inflammation and anaphylaxis, the results may not directly mimic effects in other animal species or human inflammatory disease [88, 89]. Predictions about human health will, therefore, require validation through further experimentation and/or clinical trials.

Based on our data, the observed HFE antihistaminic activities are expected to be mediated in part through interaction with the H1R and H4R receptors, both of which play important roles in the progression and modulation of histamine-mediated conditions [90–92]. It is considered also that the absence of acute toxicity at 5000 mg/kg bw adds to the evidence for usefulness of HFE. Further, having both prophylactic and therapeutic inhibitory activities suggests that HFE is capable of interaction with other key mediators of mechanisms that underlie different pathways of inflammation development. Specific investigations into HFE interaction with signalling intermediates pertinent to modulation of the inflammatory response, including relevance to COVID-19, are warranted. Some anti-inflammatory agents, including dexamethasone, hydroxychloroquine and famotidine, have shown utility in COVID-19 management. Famotidine is an anti-inflammatory antihistamine whose usefulness against COVID-19 is evidently not achieved by antiviral activity [63, 93] Hydroxychloroquine on the other hand is a DMARD whose usefulness in COVID-19 management is suggested to be both anti-inflammatory and antiviral [94, 95].

## Conclusion

HFE has broad-spectrum anti-inflammatory activity, as seen by its comparable activity to the several agents used as standards in this study. The data provide evidence that HFE has anti-anaphylactic and anti-oedematogenic effects: possibly through interference with histamine, serotonin and prostaglandin E_2_ signalling, as well as interaction with several other factors which mediate development and progression of inflammation. Inquiry to elucidate the mechanism(s) of action of HFE is warranted.

## Data Availability

The datasets used and/or analysed during the current study are available from the corresponding author on reasonable request.

## Abbreviations

HFE: *Holarrhena floribunda* Hydro ethanol extract
DMARDs: Disease-modifying anti rheumatic drugs
BSA: Bovine serum albumin
PBS: Phosphate buffered saline
SDR: Sprague Dawley rat
CPMR: Centre for Plant Medicine Research
GAFCO: Ghana Agro Food Company
ASA: Acetyl salicylic acid
HR: Histamine receptor
ANOVA: Analysis of variance
COX: Cyclooxygenase
AUC: Area under the curve

## References

[1] Girija ASS, Shankar EM, Larsson M (2020). Could SARS-CoV-2-Induced Hyperinflammation Magnify the Severity of Coronavirus Disease (CoViD-19) Leading to Acute Respiratory Distress Syndrome?. Front Immunol.

[2] Song P, Li W, Xie J, Hou Y, You C (2020). Cytokine storm induced by SARS-CoV-2. Clin Chim Acta.

[3] Ye Q, Wang B, Mao J (2020). The pathogenesis and treatment of the ‘Cytokine Storm’’ in COVID-19’. J Infect.

[4] Pahwa R, Goyal A, Jialal I. Chronic Inflammation. Pathobiology of Human Disease: A Dynamic Encyclopedia of Disease Mechanisms. 2021. p. 300–14.

[5] Roth GA, Abate D, Abate KH, Abay SM, Abbafati C, Abbasi N (2018). Global, regional, and national age-sex-specific mortality for 282 causes of death in 195 countries and territories, 1980–2017: a systematic analysis for the Global Burden of Disease Study 2017. The Lancet.

[6] Straub RH, Schradin C (2016). Chronic inflammatory systemic diseases – an evolutionary trade-off between acutely beneficial but chronically harmful programs. Evol Med Public Health.

[7] Minozzi S, Bonovas S, Lytras T, Pecoraro V, González-Lorenzo M, Bastiampillai AJ (2016). Risk of infections using anti-TNF agents in rheumatoid arthritis, psoriatic arthritis, and ankylosing spondylitis: A systematic review and meta-analysis. Opin Drug Safety.

[8] de Nard F, Todoerti M, Grosso Rossi, Monti S, Breda S, Rossi S (2015). Risk of hepatitis B virus reactivation in rheumatoid arthritis patients undergoing biologic treatment: Extending perspective from old to newer drugs. World J Hepatol.

[9] Lieberman P, Nicklas RA, Oppenheimer J, Kemp SF, Lang DM, Bernstein DI (2010). The diagnosis and management of anaphylaxis practice parameter: 2010 Update. J Allergy Clin Immunol.

[10] Brown A (2009). Current management of anaphylaxis. Emergencias.

[11] Laine L, Smith R, Min K, Chen C, Dubois RW (2006). Systematic review: The lower gastrointestinal adverse effects of non-steroidal anti-inflammatory drugs. Aliment Pharmacol Ther.

[12] Thomford NE, Senthebane DA, Rowe A, Munro D, Seele P, Maroyi A (2018). Natural Products for Drug Discovery in the 21st Century: Innovations for Novel Drug Discovery. Int J Mol Sci.

[13] Atanasov AG, Waltenberger B, Pferschy-Wenzig E-M, Linder T, Wawrosch C, Uhrin P (2015). Discovery and resupply of pharmacologically active plant-derived natural products: A review. Biotechnol Adv.

[14] Cragg GM, Newman DJ (2013). Natural products: A continuing source of novel drug leads. Biochim Biophys Acta Gen Subj.

[15] Pan SY, Zhou SF, Gao SH, Yu ZL, Zhang SF, Tang MK (2013). New perspectives on how to discover drugs from herbal medicines: CAM’S outstanding contribution to modern therapeutics. Evidence-based Complement Altern Med.

[16] Gnangoran B, N’Guessan B, Amoateng P, Dosso K, Yapo A, Ehile EE (2012). Hypoglycaemic activity of ethanolic leaf extract and fractions of Holarrhena floribunda (Apocynaceae). J Med Biomedi Sci.

[17] Chukwurah BKC (1997). Antimicrobial activity of Holarrhena floribunda stem bark ethanol extract. Fitoterapia.

[18] Morris C (2003). Carrageenan-Induced Paw Edema in the Rat and Mouse. Methods Mol Biol.

[19] Salvemini D, Wang ZQ, Wyatt PS, Bourdon DM, Marino MH, Manning PT (1996). Nitric oxide: A key mediator in the early and late phase of carrageenan-induced rat paw inflammation. Br J Pharmacol.

[20] Vinegar R, Schreiber W, Hugo R (1969). Biphasic development of carrageenin edema in rats. J Pharmacol Exper Ther.

[21] Crunkhorn P, Meacock SC (1971). Mediators of the inflammation induced in the rat paw by carrageenin. Br J Pharmacol.

[22] Lasselin J, Schedlowski M, Karshikoff B, Engler H, Lekander M, Konsman JP (2020). Comparison of bacterial lipopolysaccharide-induced sickness behavior in rodents and humans: Relevance for symptoms of anxiety and depression. Neurosci Biobehav Rev.

[23] Strait RT, Morris SC, Yang M, Qu XW, Finkelman FD (2002). Pathways of anaphylaxis in the mouse. J Allergy Clin Immunol.

[24] Ullman-Culleré MH, Foltz CJ (1999). Body condition scoring: a rapid and accurate method for assessing health status in mice. Lab Anim Sci.

[25] Franco NH, Correia-Neves M, Olsson IAS (2012). How “humane” is your endpoint? Refining the science-driven approach for termination of animal studies of chronic infection. PLoS Pathog.

[26] Ray MA, Johnston NA, Verhulst S, Trammell RA, Toth LA (2010). Identification of markers for imminent death in mice used in longevity and aging research. J Am Assoc Lab Anim Sci.

[27] Zatroch KK, Knight CG, Reimer JN, Pang DSJ (2017). Refinement of intraperitoneal injection of sodium pentobarbital for euthanasia in laboratory rats (Rattus norvegicus). BMC Vet Res.

[28] Church MK, James GWL, Miller P (1974). The inhibitory effect of insulin on pinnal anaphylaxis in the mouse. BRITJPHARMACOL.

[29] Kim SH, Choi CH, Kim SY, Eun JS, Shin TY (2005). Anti-allergic effects of Artemisia iwayomogi on mast cell-mediated allergy model. Exp Biol Med.

[30] Lowry SF (2005). Human endotoxemia: A model for mechanistic insight and therapeutic targeting. Shock.

[31] Abe H, Katada K, Orita M, Nishikibe M (1991). Effects of calcium antagonists on the erythrocyte membrane. J Pharm Pharmacol.

[32] Shinde UA, Phadke AS, Nair AM, Mungantiwar AA, Dikshit VJ, Saraf MN (1999). Membrane stabilizing activity — a possible mechanism of action for the anti-inflammatory activity of Cedrus deodara wood oil. Fitoterapia.

[33] Ferré S, Guix T, Prat G, Jane F, Casas M (1990). Is experimental catalepsy properly measured?. Pharmacol Biochem Behav.

[34] Winter CA, Risley EA, Nuss GW (1962). Carrageenin-Induced Edema in Hind Paw of the Rat as an Assay for Antiinflammatory Drugs. Proc Soc Exp Biol Med.

[35] Singh RK, Pandey BL (1996). Anti-inflammatory activity of seed extracts of Pongamia pinnata in rat. Indian J Physiol Pharmacol.

[36] Waalkes TP, Coburn H (1960). The role of histamine and serotonin during anaphylaxis in the mouse. J Allergy.

[37] White MV (1990). The role of histamine in allergic diseases. J Allergy Clin Immunol.

[38] Evans H, Killoran KE, Mitre E (2014). Measuring local anaphylaxis in mice. J Vis Exp.

[39] Bryce PJ, Falahati R, Kenney LL, Leung J, Bebbington C, Tomasevic N (2016). Humanized mouse model of mast cell–mediated passive cutaneous anaphylaxis and passive systemic anaphylaxis. J Allergy Clin Immunol.

[40] Ennis M, Pearce FL, Weston PM (1980). Some studies on the release of histamine from mast cells stimulated with polylysine. Br J Pharmacol.

[41] Chatterjea D, Wetzel A, Mack M, Engblom C, Allen J, Mora C (2012). Mast cell degranulation mediates compound 48/80-induced hyperalgesia in mice. Biochem Biophys Res Commun.

[42] Juskewitch JE, Knudsen BE, Platt JL, Nath KA, Knutson KL, Brunn GJ (2012). LPS-induced murine systemic inflammation is driven by parenchymal cell activation and exclusively predicted by early MCP-1 plasma levels. Am J Pathol.

[43] Lee J-K, Lee S, Baek M-C, Lee B-H, Lee H-S, Kwon TK (2017). Association between perfluorooctanoic acid exposure and degranulation of mast cells in allergic inflammation. J Appl Toxicol.

[44] Tasaka K, Mio M, Okamoto M (1986). Intracellular calcium release induced by histamine releasers and its inhibition by some antiallergic drugs. Ann Allergy.

[45] Schemann M, Kugler EM, Buhner S, Eastwood C, Donovan J, Jiang W (2012). The Mast Cell Degranulator Compound 48/80 Directly Activates Neurons. PLoS One.

[46] Reimann T,, Büscher D, Hipskind RA, Krautwald S, Lohmann-Matthes ML, Baccarini  M (1994). Lipopolysaccharide induces activation of the Raf-1/MAP kinase pathway. A putative role for Raf-1 in the induction of the IL-1 beta and the TNF-alpha genes. J Immunol (Baltimore, Md: 1950).

[47] Shapira L, Soskolne WA, Houri Y, Barak V, Halabi A, Stabholz A (1996). Protection against endotoxic shock and lipopolysaccharide-induced local inflammation by tetracycline: correlation with inhibition of cytokine secretion. Infect Immun.

[48] Qiao H, Andrade MV, Lisboa FA, Morgan K, Beaven MA (2006). FcϵR1 and toll-like receptors mediate synergistic signals to markedly augment production of inflammatory cytokines in murine mast cells. Blood.

[49] Yang C, Mo X, Lv J, Liu X, Yuan M, Dong M (2012). Lipopolysaccharide enhances FcɛRI-mediated mast cell degranulation by increasing Ca2+ entry through store-operated Ca2+ channels: implications for lipopolysaccharide exacerbating allergic asthma. Exp Physiol.

[50] Perez EM, Rogers LK, Smith CV, Weisman LE (1998). Pharmacokinetics of Dexamethasone in Rats 346. Pediatr Res.

[51] Savard C, Lema PP, Hélie P, Vachon P (2009). Effects of timing of dexamethasone treatment on the outcome of collagenase-induced intracerebral hematoma in rats. Comp Med.

[52] Hirasawa N, Murakami A, Ohuchi K (2001). Expression of 74-kDa histidine decarboxylase protein in a macrophage-like cell line RAW 264.7 and inhibition by dexamethasone. Eur J Pharmacol.

[53] Hirasawa N (2019). Expression of Histidine Decarboxylase and Its Roles in Inflammation. Int J Mol Sci.

[54] Earp JC, Pyszczynski NA, Molano DS, Jusko WJ (2008). Pharmacokinetics of dexamethasone in a rat model of rheumatoid arthritis. Biopharm Drug Dispos.

[55] Kelmenson AT, Rao NK, Raizman MB. 17 - Treatment of Allergic Eye Disease. In: Holland EJ, Mannis MJ, Lee Conjunctiva and Tear Film WBBT-OSDC, editors. London: W.B. Saunders; 2013. p. 117–24.

[56] Pearce FL (1985). Calcium and mast cell activation. British journal of clinical pharmacology.

[57] Sanberg PR (1980). Haloperidol-induced catalepsy is mediated by postsynaptic dopamine receptors. Nature.

[58] Stanworth DR. Immediate hypersensitivity: the molecular basis of the allergic response. Amsterdam: North-Holland Publishing Company; 1973. p. 73.

[59] Lakdawala AD, Dadkar NK, Dohadwalla AN (1980). Action of clonidine on the mast cells of rats. J Pharm Pharmacol.

[60] Chopra YM, Dandiya PC (1975). The relative role of brain acetylcholine and histamine in perphenazine catatonia and influence of antidepressants and diphenhydramine alone and in combination. Neuropharmacology.

[61] Jadhav JH, Balsara JJ, Chandorkar AG (1983). Involvement of histaminergic mechanisms in the cataleptogenic effect of clonidine in mice. J Pharm Pharmacol.

[62] Cowden JM, Zhang M, Dunford PJ, Thurmond RL (2010). The Histamine H_4_ Receptor Mediates Inflammation and Pruritus in Th2-Dependent Dermal Inflammation. J Investig Dermatol.

[63] Malone RW, Tisdall P, Fremont-Smith P, Liu Y, Huang XP, White KM (2021). COVID-19: Famotidine, Histamine, Mast Cells, and Mechanisms. Front Pharmacol..

[64] Huang C, Wang Y, Li X, Ren L, Zhao J, Hu Y (2020). Clinical features of patients infected with 2019 novel coronavirus in Wuhan. China The Lancet.

[65] Ruan Q, Yang K, Wang W, Jiang L, Song J (2020). Clinical predictors of mortality due to COVID-19 based on an analysis of data of 150 patients from Wuhan. China Intensive Care Medicine.

[66] Zhang C, Wu Z, Li J-W, Zhao H, Wang G-Q (2020). Cytokine release syndrome in severe COVID-19: interleukin-6 receptor antagonist tocilizumab may be the key to reduce mortality. Int J Antimicrob Agents.

[67] Branco ACCC, Yoshikawa FSY, Pietrobon AJ, Sato MN (2018). Role of Histamine in Modulating the Immune Response and Inflammation. Mediators Inflamm.

[68] Winbery SL, Lieberman PL (2002). Histamine and antihistamines in anaphylaxis. Clin Allergy Immunol.

[69] Hill SJ (1992). Multiple histamine receptors: properties and functional characteristics. Biochem Soc Trans.

[70] Barnes PJ (1991). Histamine receptors in the lung. Agents Actions.

[71] Kritas SK, Ronconi G, Caraffa A, Gallenga CE, Ross R, Conti P. Mast cells contribute to coronavirus-induced inflammation: new anti-inflammatory strategy. Vol. 34, Journal of biological regulators and homeostatic agents. Italy; 2020. p. 9–14. 10.23812/20-Editorial-Kritas 32013309

[72] Kilinc E, Baranoğlu Y, Baranoğlu KY (2020). Mast cell stabilizers as a supportive therapy can contribute to alleviate fatal inflammatory responses and severity of pulmonary complications in COVID-19 infection. Anadolu Kliniği Tıp Bilimleri Dergisi.

[73] Zwickl H, Zwickl-Traxler E, Pecherstorfer M (2019). Is Neuronal Histamine Signaling Involved in Cancer Cachexia? Implications and Perspectives. Front Oncol.

[74] Becker S, Pflugbeil C, Gröger M, Canis M, Ledderose GJ, Kramer MF (2012). Olfactory dysfunction in seasonal and perennial allergic rhinitis. Acta Otolaryngol.

[75] Lechien JR, Chiesa-Estomba CM, Place S, Van Laethem Y, Cabaraux P, Mat Q, et al. Clinical and epidemiological characteristics of 1420 European patients with mild-to-moderate coronavirus disease 2019. Journal of Internal Medicine. 2020 Apr 30;n/a(n/a). 10.1111/joim.13089 PMC7267446 32352202

[76] Geurdes H. Hypothesis Why Vertical SARS-CoV-2 Infection Can Occur and Is Rare. SSRN Electronic Journal. 2020;

[77] Aydin S, Aydin S. Could Antihistamines Help in the Treatment and Spread of COVID-19 Via Re-Modulating Cytokines and by Reducing Sneezing? 2020 27;

[78] Posadas I, Bucci M, Roviezzo F, Rossi A, Parente L, Sautebin L (2004). Carrageenan-induced mouse paw oedema is biphasic, age-weight dependent and displays differential nitric oxide cyclooxygenase-2 expression. Br J Pharmacol.

[79] Lo TN, Saul WF, Lau SS (1987). Carrageenan-stimulated release of arachidonic acid and of lactate dehydrogenase from rat pleural cells. Biochem Pharmacol.

[80] Hwang SB, Lam MH, Li CL, Shen TY. Release of platelet activating factor and its involvement in the first phase of carrageenin-induced rat foot edema. Eur J Pharmacol. 1996;33(5):693–701. 10.1016/0014-2999(86)90636-9 3948914

[81] Nantel F, Denis D, Gordon R, Northey A, Cirino M, Metters KM (1999). Distribution and regulation of cyclooxygenase-2 in carrageenan-induced inflammation. Br J Pharmacol.

[82] Gupta M, Mazumder UK, Gomathi P, Selvan VT (2006). Antiinflammatory evaluation of leaves of Plumeria acuminata. BMC Complement Altern Med.

[83] Gan TJ (2010). Diclofenac: an update on its mechanism of action and safety profile. Curr Med Res Opin.

[84] Scholer DW, Ku EC, Boettcher I, Schweizer A (1986). Pharmacology of diclofenac sodium. Am J Med.

[85] Voilley N, de Weille J, Mamet J, Lazdunski M (2001). Nonsteroid anti-inflammatory drugs inhibit both the activity and the inflammation-induced expression of acid-sensing ion channels in nociceptors. J Neurosci.

[86] Kaibara N, Hotokebuchi T, Takagishi K, Katsuki I (1983). Paradoxical effects of cyclosporin A on collagen arthritis in rats. J Exp Med.

[87] Larsson P, Kleinau S, Holmdahl R, Klareskog L (1990). Homologous type II collagen-induced arthritis in rats. Characterization of the disease and demonstration of clinically distinct forms of arthritis in two strains of rats after immunization with the same collagen preparation.. Arthritis Rheumatism.

[88] Seok J, Warren HS, Cuenca AG, Mindrinos MN, Baker HV, Xu W (2013). Genomic responses in mouse models poorly mimic human inflammatory diseases. Proc Natl Acad Sci USA.

[89] Burkhardt AM, Zlotnik A (2013). Translating translational research: mouse models of human disease. Cell Mol Immunol.

[90] Jemima EA, Prema A, Thangam EB (2014). Functional characterization of histamine H4 receptor on human mast cells. Mol Immunol.

[91] Ohsawa Y, Hirasawa N (2014). The role of histamine H1 and H4 receptors in atopic dermatitis: from basic research to clinical study. Allergology Int.

[92] Thangam EB, Jemima EA, Singh H, Baig MS, Khan M, Mathias CB (2018). The Role of Histamine and Histamine Receptors in Mast Cell-Mediated Allergy and Inflammation: The Hunt for New Therapeutic Targets. Front Immunol.

[93] Freedberg DE, Conigliaro J, Wang TC, Tracey KJ, Callahan MV, Abrams JA (2020). Famotidine Use is Associated with Improved Clinical Outcomes in Hospitalized COVID-19 Patients: A Propensity Score Matched Retrospective Cohort Study. Gastroenterology.

[94] Liu J, Cao R, Xu M, Wang X, Zhang H, Hu H (2020). Hydroxychloroquine, a less toxic derivative of chloroquine, is effective in inhibiting SARS-CoV-2 infection in vitro. Cell discovery.

[95] Ornstein MH, Sperber K (1996). The antiinflammatory and antiviral effects of hydroxychloroquine in two patients with acquired immunodeficiency syndrome and active inflammatory arthritis. Arthritis Rheum.

